# Disclosure of Mental Health Status to Employers in a Healthcare Context

**DOI:** 10.21315/mjms2021.28.2.14

**Published:** 2021-04-21

**Authors:** Yusrita Zolkefli

**Affiliations:** PAPRSB Institute of Health Sciences, Universiti Brunei Darussalam, Brunei Darussalam

**Keywords:** disclosure, mental health, status, ethics, health professionals

## Abstract

People suffering from mental health conditions are often unwilling to reveal their status and this includes health professionals. They may wrestle with the pros and cons of revealing their health status to their employer in particular as they seek to reconcile personal privacy with professional duty. There is no simple, clear consensus as to whether they have a moral duty to share the information voluntarily or explicitly to share it with the employer. Additionally, there is a concern as to whether a degree of non-disclosure is justifiable to protect the privacy of health care professionals in some circumstances. Decisions surrounding the disclosure of a mental health problem are nuanced and may require that competing needs and values be reconciled. Although self-declared mental health status is an intrinsic moral good, the healthcare professional needs to feel confident and ready to come forward.

## Introduction

Healthcare professionals’ mental health is particularly important because poor mental health is linked to medical errors (1–2) and decreased performance (3), which could harm the health of patients. Mental health issues faced by healthcare staff also lead to a high turnover rate (4) which increases medical institutions’ costs through training expenses and decreased efficiency (5). Combined, these consequences threaten patient safety. The World Health Organization (6) has estimated that the overall number of people with depression in the world in 2015 reached 300 million and that a comparable number of people have anxiety disorders. However, people with these disorders are often reluctant to reveal their condition (7–9). This includes healthcare professionals. They may be struggling with the pros and cons of disclosing their health status, especially to their employer, as they try to balance personal privacy with professional duty.

## The Uncertainty of Disclosure

There is no clear, unequivocal consensus on whether they have a moral obligation to share the information voluntarily in general or specifically to share it with the employer. In addition, there is uncertainty as to whether a degree of non-disclosure is justifiable in certain situations to protect the privacy of the healthcare professionals. A dichotomous definition of disclosure (i.e. disclosure versus non-disclosure) is insufficient for characterising the nuances involved in the process. In a systematic review of beliefs, behaviours and influencing factors associated with disclosure of a mental health problem in the workplace (7), four dimensions of disclosure were discussed: i) voluntary or involuntary (disclosure is either controlled by the individuals affected or is visible in speech, behaviour, or appearance); ii) full or partial (which aspects of the condition should be disclosed); iii) selectivity (e.g. whether to disclose widely or only to select individuals) and iv) the timing of the disclosure.

It must also be remembered that the personal health of a healthcare professional is often considered private and without the confidentiality offer by the employer or the workplace organisation, the healthcare professionals may hesitate to disclose their mental health status. The systematic review revealed that the attitudes associated with mental illness in employees are mostly negative, and, in turn, the behaviours that co-workers and supervisors direct towards mentally ill individuals often lead to both open and subtle discrimination, reducing the quality of the work experience for those individuals (10). Furthermore, since personal control is considered an intrinsic and central social need (11) individuals have a psychological need to control when and how they reveal their mental health status. Concealment can offer particular psychological benefits for individuals who are dispositionally concerned with social rejection (12). Healthcare practitioners also face the additional concern that a diagnosis of mental illness could contribute to an investigation of their ability to practice and may harm the future of a healthcare professional, for example, by damaging their professional credibility, professional accreditation, career advancement or job status, to the point of leading to dismissal (13).

## A Win-Win Situation

For a win-win situation, both the employees and the employers have shared duties to discharge. Healthcare professionals have a stronger professional and ethical duty not to harm patients. Most ethical codes of ethics and professional conduct guidelines establish that healthcare professionals must make the care of patients and colleagues their first concern, and to practice safely and effectively. That means that they are responsible for protecting and supporting the health of individuals. It may also be necessary to alert the employer when they become aware of a health problem that could prevent them from working. Furthermore, healthcare workers with mental illness must disclose their mental illness at work in order for them to get ‘work accommodation’. In most cases, this allows employers to assist the healthcare professional with adequately limiting or discontinuing practices that might harm patients, while taking appropriate steps to address the impairment. Simultaneously, employers have a duty of care to their workers. This means that employers have to ensure that workers work in a healthy atmosphere and have the necessary resources and incentives to help them preserve their health, well-being, and safety wherever they operate.

Equally fundamental is an adequately robust policies and procedures in place, such as safeguarding the confidentiality of healthcare professionals. A recent study asked stakeholders about their views on the advantages and disadvantages of divulging a mental illness in a general workplace such as companies in consultancy, catering, cleaning, construction, and the plastics industry (14). All groups consisted of people with mental illness, human resources managers, involved in hiring decisions, employers, work reintegration professionals and mental health advocates agreed that disclosure increases understanding of the actions and condition of the disclosers, and therefore, can strengthen relationships at work. Furthermore, under the theme of ‘being allowed to be who you are (authenticity) is important for well-being at work’, three groups consisted of people with mental illness, advocates and professionals agreed that concealing mental illness or mental health issues would make people feel dishonest, guilty and tired.

Disclosure unfortunately exposes people to the risk of stigma and discriminatory treatment (15), and these two factors act as a barrier to finding and maintaining work for mentally ill individuals (7). The dismissive attitudes of managers towards those experiencing mental illness at work often contribute to an inability to help with their employees’ mental health, even given the fact that workers become sick or that their symptoms worsen while on duty (16). The responses of managers to workplace mental illness are often paternalistic and protective, and the responsibilities of the organisations are reduced to doing only what is required by law or by internal policies and procedures. Others have also suggested that employers need to learn more about mental illness in the workplace (17–19) and that this may reduce workplace mental health stigma (18, 20). Helping people through the disclosure process can also be beneficial (21). Such help can be in the form of peer support for mental health as a way of countering perceived stigma. The same weight of support can be develop through a non-stigmatic relationship with people who have similar experiences.

## The Way Forward

To summarise, decisions regarding the disclosure of a mental health issue are complex and may require the reconciliation of conflicting needs and values. While self-declared mental health status is an essential moral good, the healthcare professionals must feel confident and ready to come forward. It does not, however, imply that, just because it is a personal choice to reveal or not, the status can be swept under the rug and dismiss the impact of non-disclosure. If the employers are unaware of such status, it will also be unreasonable to be expecting the employer to provide support in a timely and appropriate manner.

While employers are justified in requiring the healthcare professionals to disclose their mental health status, it must be assured that this private information is kept confidential. The stigma attached to particular illnesses is not easy to overcome. Nonetheless, the employer must, first and foremost, be transparent, sensitive, and trustworthy when such disclosure is expected, through robust and prompt measures in responding to every situation of a healthcare professional who may potentially has mental illness. The attention should also be on accommodating the health needs of healthcare professionals and established that both the employer and employee have reciprocal professional and ethical commitments to create a healthy, safe and productive workplace.
